# Cosmc loss induces truncated O-glycosylation and accelerates Kras-driven pancreatic carcinogenesis

**DOI:** 10.1016/j.neo.2026.101347

**Published:** 2026-07-31

**Authors:** Baris Mercanoglu, Nina Schraps, Anastasios D. Giannou, Jingxuan Zhou, Simon Kind, Maya Reinecke, Karl-Frederick Karstens, Benjamin Dreyer, Sönke Harder, Siwen Zhang, Eleftherios D. Papazoglou, Cenap Güngör, Nathaniel Melling, Hartmut Schlüter, Christoph Wagener, Maximilian Bockhorn, Thilo Hackert, Gerrit Wolters-Eisfeld

**Affiliations:** a Department of General, Visceral and Thoracic Surgery, University Medical Center Hamburg-Eppendorf, Hamburg, Germany; b Section of Molecular Immunology und Gastroenterology, I. Department of Medicine, University Medical Center Hamburg-Eppendorf, Hamburg, Germany; c Hamburg Center for Translational Immunology (HCTI), University Medical Center Hamburg-Eppendorf, Hamburg, Germany; d Department of Surgery, Division of General Surgery, The University of British Columbia, Vancouver, Canada; e Department of Urologic Sciences, The University of British Columbia, Vancouver, Canada; f Department of Pathology, University Medical Center Hamburg-Eppendorf, Hamburg, Germany; g Mass Spectrometric Proteomics - Institute for Clinical Chemistry & Laboratory Medicine, University Medical Center Hamburg-Eppendorf, Hamburg, Germany; h Medical Faculty, Universität Hamburg, Hamburg, Germany; i Department of General and Visceral Surgery, University Medical Center Oldenburg, Oldenburg, Germany

**Keywords:** Pancreatic cancer, PDAC, Cosmc, O-glycosylation, Tn antigen, Kras, Mouse model

## Abstract

Aberrant mucin-type O-glycosylation is a common feature of pancreatic ductal adenocarcinoma (PDAC), yet its functional contribution to pancreatic tumorigenesis remains incompletely defined. In particular, whether defective core 1 O-glycan maturation cooperates with oncogenic KRAS during early pancreatic neoplasia has not been fully resolved. Here, we addressed this question by deleting *Cosmc* (*C1galt1c1*), the obligate molecular chaperone for T-synthase (*C1galt1*), in a pancreas-specific *Kras*-driven mouse model. Analysis of human PDAC tissue microarrays revealed frequent expression of the truncated O-glycan epitopes Tn and sialyl-Tn, supporting the clinical relevance of impaired O-glycan elongation in pancreatic cancer. *In vivo*, pancreas-specific *Cosmc* loss in the setting of oncogenic *Kras* accelerated pancreatic tumor progression, enhanced tissue proliferation, altered acidic mucin-associated glycosylation, and promoted pronounced stromal remodeling. Lectin histochemistry and biochemical analyses confirmed robust accumulation of Tn antigen in *Cosmc*-deficient pancreatic tumors. To define molecular programs associated with this phenotype, we performed transcriptomic and proteomic profiling of tumor-derived PDAC cell lines together with VVA-enriched glycoproteomic analysis of pancreatic tissues and cell lines. These approaches identified coordinated remodeling of pathways linked to extracellular matrix organization, adhesion, cytoskeletal regulation, stress adaptation, and metabolic reprogramming. Together, these data identify *Cosmc*-dependent O-glycosylation as a regulator of *Kras*-driven pancreatic carcinogenesis. Our findings support a model in which truncated O-glycans contribute to PDAC progression in association with remodeling of the tumor glycoproteome and stromal architecture during *Kras*-driven pancreatic tumor evolution.

## Highlights

• Tn antigen is frequently expressed in human PDAC and associates with tumor stage.
• Cosmc loss accelerates early Kras-driven pancreatic neoplastic progression.
• Cosmc-deficient tumors show increased fibrosis, proliferation, and altered mucin-associated glycosylation.
• Cosmc loss induces a robust Tn-like glycoprotein phenotype in pancreatic tumors.
• Glycoproteomic profiling links Cosmc loss to extracellular matrix and stromal remodeling.

## Introduction

Pancreatic ductal adenocarcinoma (PDAC) remains among the deadliest human malignancies, with poor long-term survival, rising incidence and limited therapeutic options [1,2]. Most patients are diagnosed at an advanced stage, when curative treatment is no longer feasible and systemic therapies provide only modest benefit [3,4]. PDAC develops through recognizable precursor lesions and is driven by recurrent alterations in *KRAS, TP53, CDKN2A* and *SMAD4*, with oncogenic *KRAS* acting as a central initiating event [[5], [6], [7], [8]]. Defining molecular mechanisms that cooperate with KRAS during early neoplastic progression therefore remains a central challenge in pancreatic cancer biology [9].

Beyond genetic alterations, post-translational mechanisms increasingly emerge as active regulators of tumor behavior [10,11]. Mucin-type O-GalNAc glycosylation is particularly relevant in epithelial cancers, where it regulates mucin function, adhesion, receptor signaling and immune recognition [12]. In malignant cells, impaired elongation of core 1 O-glycans frequently results in accumulation of truncated structures such as the Tn antigen and its sialylated derivative, sialyl-Tn [13]. These immature glycoforms can arise from defective C1GALT1/T-synthase activity or disruption of its obligate endoplasmic reticulum chaperone COSMC/C1GALT1C1 [14]. Tn and sialyl-Tn antigens are widely detected in human PDAC [15,16], yet whether truncated O-glycans functionally contribute to pancreatic tumor initiation and early progression remains incompletely understood.

*COSMC* loss provides a genetically defined mechanism to induce persistent O-glycan truncation and Tn-dominant glycosylation [11,14,17,18]. Prior work from our group showed that *COSMC* depletion promotes oncogenic properties in pancreatic cancer cell lines [10]. We also previously generated and characterized the pancreas-specific *Ptf1a*-Cre;*Cosmc*^fl/fl^ model in the absence of oncogenic *Kras*. In this model, *Cosmc* deletion induced Tn and sialyl-Tn dominant O-glycosylation in pancreatic acinar cells and caused impaired digestive enzyme activity and secretion, zymogen granule accumulation, exocrine pancreatic insufficiency, age-dependent pancreatic atrophy, and disturbances in glucose homeostasis. No pancreatic neoplastic phenotype was observed, suggesting that defective O-glycan elongation disrupts pancreatic physiology but is not sufficient to initiate tumorigenesis in this model [18]. However, whether defective core 1 O-glycan maturation cooperates with oncogenic *Kras* to accelerate early pancreatic neoplasia *in vivo* remains unresolved.

Genetically engineered mouse models have been instrumental in defining PDAC initiation and progression [19,20]. Because KC mice progress relatively slowly, this model allows early epithelial and stromal effects of *Cosmc* loss to be resolved before the emergence of highly aggressive disease [[21], [22], [23]]. This distinction is important because early epithelial and stromal effects caused by an additional modifier may be difficult to resolve in an aggressive KPC setting. A *Kras*-only background therefore provides a tractable framework to test whether truncated O-glycans are sufficient to enhance early neoplastic progression, including effects on precursor lesion development, epithelial proliferation, mucin remodeling and stromal evolution [24,25]. Consistent with the relevance of this pathway, C1galt1 deletion in KPC mice was previously shown to accelerate PanIN development and metastatic progression [26].

Here, we investigate the functional consequences of *Cosmc* loss and truncated O-glycosylation in *Kras*-driven pancreatic tumorigenesis. Using human PDAC tissue microarrays, genetically engineered mouse models, lectin histochemistry, transcriptomic profiling, and VVA-enriched glycoproteomics, we show that truncated O-glycans are prevalent in human PDAC and that pancreatic epithelial *Cosmc* loss accelerates *Kras*-driven tumor progression *in vivo*. This phenotype is accompanied by robust Tn antigen accumulation, stromal remodeling, loss of acidic mucins, and glycoproteomic changes linked to extracellular matrix organization, adhesion, and stress/metabolic adaptation. These findings support a model in which disrupted mucin-type O-glycosylation contributes to pancreatic cancer progression by reshaping the tumor glycoproteome and its interaction with the stromal microenvironment.

## Materials and methods

### Animal models

Mouse models were generated on a C57BL/6J background. The *LSL-Kras^G12D^* allele (B6.129-*Kras^tm4Tyj^*, RRID:IMSR_JAX:008179) expresses oncogenic *Kras^G12D^* upon Cre-mediated excision of a loxP-flanked stop cassette. Conditional *Cosmc* knockout mice were generated by flanking exon 2 of *C1galt1c1* with loxP sites, enabling its deletion by Cre recombinase as previously described [18]. For pancreas-specific recombination, we used *Ptf1a^Cre^* (Ptf1a^tm1(cre)Hnak/RschJ^, RRID:IMSR_JAX:023329), where Cre is driven by the endogenous *Ptf1a* promoter. Mice with the *LSL-Kras^G12D/+^;Ptf1a^Cre/+^* genotype were designated KC, and those additionally harboring *Cosmc^fl/fl^* were referred to as KCC. The breeding strategies are shown in Supplementary Fig. 1. All animals were maintained under standard housing conditions with ad libitum access to food and water. Euthanasia was performed via CO₂ asphyxiation followed by cervical dislocation. Genotyping was performed using the Kappa Mouse Genotyping Hot Start Kit (PeqLab) with the following primers: *Cre*: F: ACCAGCCAGCTATCAACTCG; R: TTACATTGGTCCAGCCACC (200 bp); *Cosmc*: F: CACAGAACTCACTATCCACTAGGCATGAATACAT; R: GCTCTCCCTAAATATACAACCGATTAAGAAAGTGT (560 bp); and *Kras^G12D^*: F: CCATGGCTTGAGTAAGTCTGC; R: CGCAGACTGTAGAGCAGCG (600 bp). All procedures were approved by the Institutional Animal Care and Use Committee and performed in accordance with institutional, national, and ARRIVE guidelines.

### Generation of GEMM-derived cell lines

Primary PDAC tumors were harvested from two 12-month-old KC and KCC mice and confirmed by histopathology. Tumors were minced and digested in 0.5% collagenase type IV (Sigma-Aldrich) at 37 °C for 45 min with agitation. Cell suspensions were centrifuged (700 × g, 5 min), washed in RPMI, and plated in collagen-coated flasks (BD). Cells were cultured in RPMI 1640 with GlutaMAX (Gibco), 10% FCS, penicillin–streptomycin (200 IU/mL), gentamycin (0.1 mg/mL), transferrin (50 nM), insulin (0.01 μg/mL), EGF (0.01 μg/mL), and bFGF (0.01 μg/mL), at 37 °C with 5% CO₂. Medium was changed every 4–7 days. Cultures were maintained as adherent monolayers, passaged by trypsinization, and cryopreserved in medium with 10% DMSO. Genotyping was performed using standard primers; control gene primers: Cntrl-F, 5′-GTCGACAAGCTCATGCGGG-3′; Cntrl-R, 5′-CGCAGACTGTAGAGCAGCG-3′.

### Histological staining, immunohistochemistry and immunofluorescence

Tissue staining was performed as previously described [27]. Primary antibodies (1:100) included anti-Tn (MA1-80055, Thermo Fisher), anti-STn (ABIN356328, Antikörper Online), anti-Ki67 (ab15580, Abcam), and anti–α-SMA (M0851, DAKO). Fluorescein-labeled VVA and rhodamine-labeled PNA (Vector Laboratories) were used for immunofluorescence. For lectin histochemistry, biotinylated VVA (B-1235) or DBA (B-1035-5) (10 µg/mL) was applied and detected using streptavidin-HRP (Thermo Fisher, 1 µg/mL). Images were acquired using a Biorevo BZ-9000 microscope (Keyence). Quantification of positive staining was performed in ImageJ (v1.54 g) and expressed as % = (area of interest/reference area)  ×  100.

### Tissue microarray

A pancreatic cancer TMA comprising 233 FFPE tumor samples from patients who underwent resection between 1992 and 2011 at the University Hospital Hamburg–Eppendorf was analyzed. Ethical approval was obtained (WF-049/09, PV3652), and all procedures adhered to the Declaration of Helsinki. TMA construction was performed as previously described [28]. Tumor grading and staging followed WHO and UICC criteria. Fresh TMA sections were processed in a single batch. Antigen retrieval was performed by autoclaving slides at 121 °C for 5 min in pH 2.0 retrieval buffer (Biogenex). Sections were incubated for 60 min at 37 °C with anti-Tn (CD175; MA1-80055, Thermo Fisher, 1:150) or anti-STn (CD175s; MA1-90577, Thermo Fisher, 1:150), followed by EnVision detection (Dako). Immunoreactivity was assessed in the cytoplasm and apical membranes. Tumors with ≥10% positive, uniformly stained cells were scored as “positive”; those without detectable staining were classified as “negative.”

### Western blot analysis

For Western blotting, antibodies directed against Gapdh (sc-32233; Santa Cruz Biotechnology) and E-cadherin (#3195T; CST) were used. For hydrolysis of α2–3-, α2–6-, and α2–8-linked sialic acid residues, 10 µg of total protein was treated with α2–3,6,8-neuraminidase A (P0722; NEB) for 20 min at 37°C. Next, 10 µg/ml biotinylated VVA (B-1235-2; Vector Laboratories) or biotinylated PNA (B-1075-5) complexed with 1 µg of streptavidin-HRP (21126; Pierce, Thermo Fisher Scientific, Grand Island, NY, USA) was used for the detection of nonsialylated Tn antigen. For preparation of the cytosolic and membrane fractions, the Subcellular Fractionation Kit (78840; Pierce, Thermo Fisher Scientific, Grand Island, NY, USA) was used according to the manufacturer’s recommendations.

### Transcriptomic analysis and gene set enrichment

Total RNA was isolated from pancreatic ductal cell lines derived from KC and KCC mice and subjected to RNA sequencing by CeGaT GmbH (Tübingen, Germany). Raw count data were analyzed in R using DESeq2. Lowly expressed genes were filtered prior to normalization, and differential expression was calculated for KCC versus KC cells. Principal component analysis was performed on variance-stabilized normalized counts to visualize genotype-associated transcriptomic differences. Differentially expressed genes were defined by an absolute log₂ fold change > 1 and Benjamini–Hochberg adjusted P < 0.05. Volcano plots were generated from DESeq2 results, with selected genes labeled according to statistical significance and fold change. Gene set enrichment analysis was performed using GSEA software version 4.4.0 with MSigDB Hallmark gene sets [29,30]. Genes were ranked according to the DESeq2-derived statistic for the KCC versus KC comparison. Normalized enrichment scores were calculated to determine pathway enrichment associated with either genotype. Positive NES values indicate enrichment in KCC cells, whereas negative NES values indicate relative enrichment in KC cells. Nominal P values and false discovery rate values were reported according to the GSEA output. All RNA-sequencing data are provided in Supplementary Material 2 and representative validation of the RNA-seq findings at the protein level is shown in Supplementary Figure 6.

### O-GalNAc glycoproteome enrichment and pathway analysis

Pancreatic lysates from *Cosmc*-KO, KCC mice and KCC-derived pancreatic cancer cell lines were treated with α2–3,6,8-neuraminidase A (NEB) and subjected to *Vicia villosa* agglutinin (VVA; Vector Laboratories) pull-down to enrich proteins carrying exposed terminal GalNAc residues. Bound proteins were eluted with 200 mM GalNAc, separated by SDS–PAGE and processed by in-gel digestion after DTT reduction, iodoacetamide alkylation and trypsin digestion. Peptides were analyzed by LC–MS/MS using a Dionex UltiMate 3000 RSLCnano coupled to an Orbitrap Fusion mass spectrometer (Thermo Scientific). MS1 spectra were acquired at 120,000 resolution across m/z 400–1500, followed by data-dependent HCD MS/MS acquisition. Spectra were searched with Sequest HT in Proteome Discoverer v2.4.1.15 against the *Mus musculus* SwissProt database, allowing two missed cleavages, 10 ppm precursor mass tolerance and 0.2 Da fragment mass tolerance. Protein identifications required ≥2 unique peptides at 1% FDR.

Overlap between VVA-enriched protein sets was visualized using Venny 2.1. Functional enrichment of VVA-enriched proteins was performed using Enrichr [31], including BioPlanet 2019, Reactome Pathways 2024 and GO Biological Process 2025 libraries. The integrated enrichment analysis shown in Fig. 5E was performed with g:Profiler [32] version e114_eg62_p19_0b5bc055, including GO, KEGG and Reactome terms. Enrichment results were ranked by adjusted p value. All relevant data are provided in Supplementary Material 3.

### Statistical analyses

Each experiment was repeated at least twice. Unless otherwise noted, the data are presented as the means ± SEMs, and a two-tailed, unpaired Student’s *t*-​test was used to compare two groups of independent samples. p < 0.05 was considered statistically significant. Statistical analysis was performed with Prism 9.5.1 software (GraphPad Software, La Jolla, CA, USA). For comparisons involving categorical variables, Fisher’s exact test or χ² test was used as appropriate. Survival differences were assessed by log-rank test. For analyses involving multiple groups or time points, one-way or two-way ANOVA with appropriate post hoc correction was used where applicable. For transcriptomic analyses, p values were adjusted using the Benjamini–Hochberg method. Enrichment analyses were interpreted using tool-specific adjusted p values or FDR values.

## Results

### Tn antigen is frequently expressed in human PDAC and associates with tumor stage

To assess truncated O-glycosylation in human PDAC, we analyzed Tn (CD175) and sialyl-Tn (STn; CD175s) expression by immunohistochemistry on PDAC tissue microarrays. Tn was detected in 67% of evaluable tumors (109/163), predominantly with weak staining, while strong positivity was observed in 5% of cases (Supplementary Fig. 2). Tn expression was significantly associated with pathological tumor stage (p = 0.0055, Supplementary Tab. 1), but not with nodal status, resection margin, grade, recurrence or overall survival. STn was similarly frequent, being detected in 66% of tumors, but showed no significant clinicopathological or survival associations. These data establish the clinical prevalence of truncated O-glycan antigens in PDAC and support experimental testing of whether O-glycan truncation can modify pancreatic tumorigenesis.

### Truncated O-glycans accelerate Kras-driven pancreatic neoplastic progression *in vivo*

**Fig. 1:**
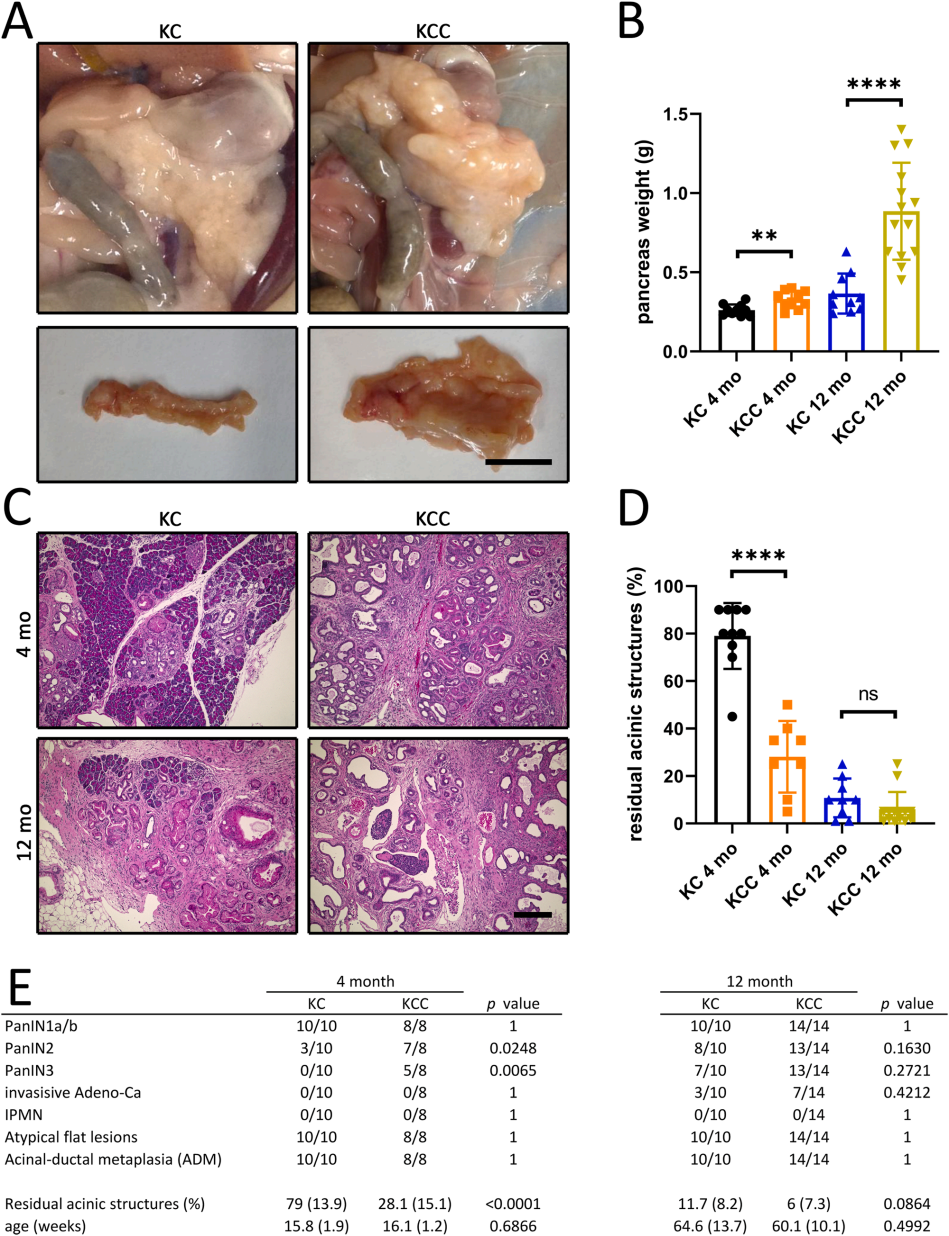
**Truncated O-glycans accelerate *Kras*-driven pancreatic tumorigenesis *in vivo***. The effect of truncated O-glycosylation on *Kras*-driven pancreatic tumorigenesis was assessed by comparing KC mice with KCC mice, in which pancreatic epithelial deletion of *Cosmc* impairs core 1 O-glycan elongation and promotes accumulation of truncated O-glycans. (A) Representative macroscopic images of pancreata from 12-month-old mice, shown *in situ* and after dissection, illustrate marked pancreatic enlargement in KCC mice compared with KC controls. Scale bar, 1 cm. (B) Pancreas weight was significantly increased in KCC mice at 4 and 12 months, indicating enhanced pancreatic tumor burden following *Cosmc* loss. n = 10 KC and n = 10 KCC mice at 4 months; n = 10 KC and n = 14 KCC mice at 12 months. Two-tailed Student’s *t*-test; p = 0.0047 and p < 0.0001. (C) Representative H&E-stained sections show advanced pancreatic remodeling in KCC mice, characterized by loss of normal acinar architecture, expansion of neoplastic epithelial lesions, and increased stromal reaction. Scale bar, 200 μm. (D) Quantification of residual acinar tissue revealed severe acinar depletion in KCC pancreata at 4 months (Two-tailed Student’s *t*-test; p < 0.0001.), consistent with accelerated replacement of exocrine tissue by neoplastic and stromal compartments. At 12 months, residual acinar tissue was low in both genotypes, with a further non-significant reduction in KCC mice. n = 10 KC and n = 8 KCC mice at 4 months; n = 9 KC and n = 14 KCC mice at 12 months. (E) Histopathological characteristics of KC and KCC pancreata at 4 and 12 months of age. Lesion incidence is presented as the number of affected animals/total number examined, whereas residual acinar structures and age are shown as mean (SD). Categorical variables were compared using Fisher’s exact test. KCC mice exhibited a higher incidence of PanIN-2 and PanIN-3 lesions at 4 months, consistent with accelerated PanIN progression.

To determine whether truncated O-glycans functionally influence *Kras*-driven pancreatic tumorigenesis, we compared pancreatic disease progression in KC (*Kras*^G12D/+^;*Ptf1a*^Cre/+^) and KCC (*Kras*^G12D/+^;*Cosmc*^fl/fl^;*Ptf1a*^Cre/+^) mice at 4 and 12 months of age. Macroscopic examination revealed more pronounced pancreatic enlargement in KCC mice compared with KC controls (Fig. 1A). Consistently, pancreatic weight was significantly increased in KCC animals at both time points, indicating accelerated disease-associated pancreatic remodeling (Fig. 1B). Histological analysis of H&E-stained sections confirmed extensive tissue remodeling in both genotypes, but KCC pancreata displayed markedly accelerated loss of normal pancreatic architecture (Fig. 1C). In particular, acinar atrophy was substantially more advanced in KCC mice at 4 months. Quantification of residual acinar tissue showed that KCC pancreata retained only 28.1% ± 15.1% acinar tissue compared with 79.0% ± 13.9% in KC controls (p < 0.0001; Fig. 1D). By 12 months, residual acinar tissue was strongly reduced in both genotypes, and the difference between KCC and KC mice was no longer statistically significant, although KCC pancreata still showed numerically lower residual acinar area (6.0% ± 7.3% vs. 11.7% ± 8.2%; p = 0.0864; Fig. 1D). These findings indicate that truncated O-glycans primarily accelerate early pancreatic tissue destruction during *Kras*-driven neoplastic progression. Successful *Cosmc* knockout was validated at the mRNA and protein levels in pancreatic tissues and tumor-derived cell lines (Supplementary Fig. 3 A and B). Western blot analysis showed a stronger phospho-p44/42 MAPK signal in representative KCC pancreatic tissues and tumor-derived cell lines than in the corresponding KC samples. Total p38 MAPK was assessed in parallel as an additional MAPK-family reference protein, and Hsp70 served as the loading control. This observation is consistent with altered ERK1/2 signaling in association with *Cosmc* loss (Supplementary Fig. 3C and D).

Low-grade lesions, including acinar-to-ductal metaplasia (ADM), atypical flat lesions, and PanIN-1a/b, were present in pancreatic tissues from both genotypes (Fig. 1E). In contrast, advanced precursor lesions were enriched in KCC mice at 4 months. PanIN-2 lesions were detected in 7 of 8 KCC mice compared with 3 of 10 KC mice (p = 0.0248), whereas PanIN-3 lesions were present in 5 of 8 KCC mice but were absent from KC mice (p = 0.0065). By 12 months, advanced PanIN lesions were common in both genotypes, and the differences were no longer statistically significant. Invasive adenocarcinoma was detected only in 12-month-old animals and occurred more frequently in KCC than in KC mice (7/14 vs. 3/10), although this difference did not reach statistical significance (p = 0.4212). No intraductal papillary mucinous neoplasms were observed. The age distribution was comparable between genotypes at both time points, supporting direct comparison of the cohorts and reducing the likelihood of age-related bias. Together, these findings demonstrate that loss of *Cosmc*-mediated O-glycan elongation accelerates *Kras*-driven pancreatic neoplastic progression *in vivo*, as reflected by early acinar loss, increased pancreatic mass, enrichment of advanced PanIN lesions, and a higher frequency of invasive carcinoma at later stages.

### Cosmc loss enhances fibrosis and proliferation and alters mucin O-glycosylation in Kras-driven pancreatic lesions

**Fig. 2:**
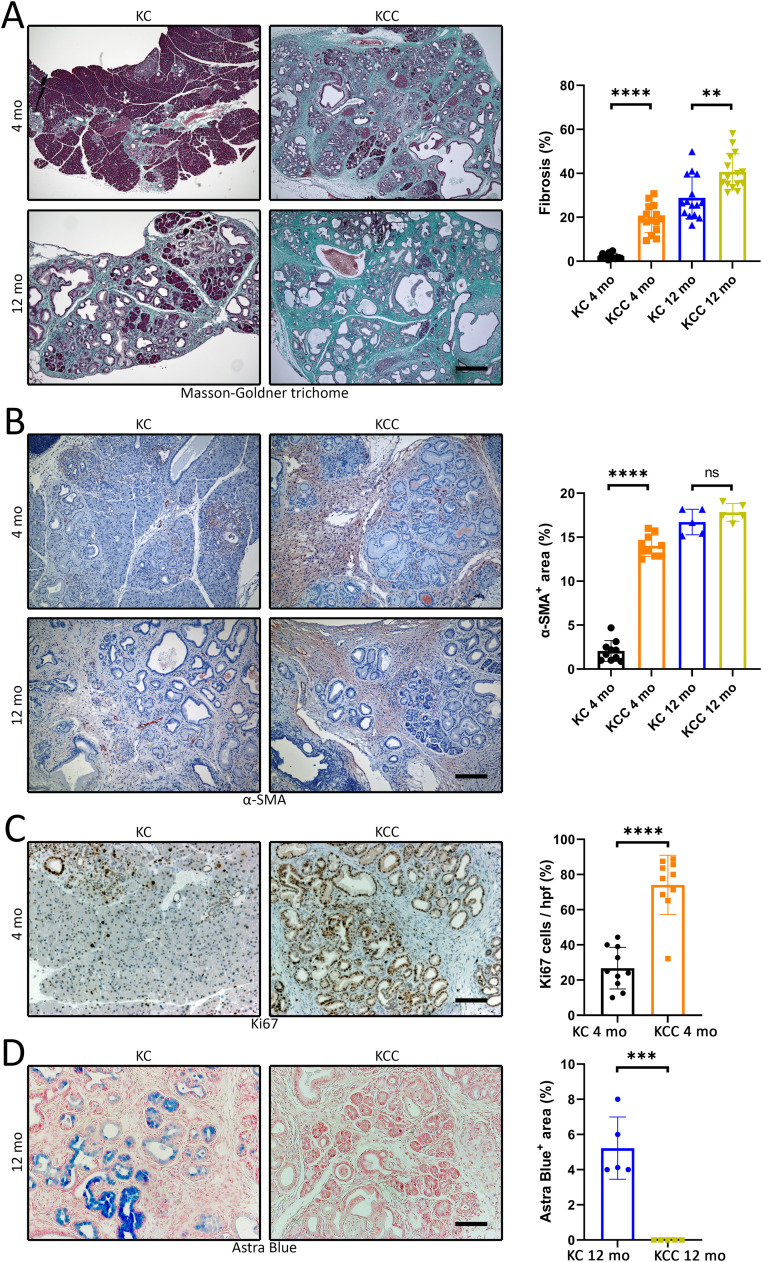
**Truncated O-glycans promote stromal remodeling, epithelial proliferation, altered acidic mucin-associated glycosylation in*****Kras*****-driven pancreatic lesions.** To assess whether accelerated tumor progression in KCC mice was accompanied by remodeling of the pancreatic tumor microenvironment, stromal deposition, fibroblast activation, epithelial proliferation, and mucin production were analyzed in KC and KCC pancreata. (A) Masson–Goldner trichrome staining revealed increased collagen-rich stromal deposition in KCC pancreata compared with KC controls. This difference was pronounced at 4 months and remained significant at 12 months, indicating enhanced fibrotic remodeling following loss of *Cosmc*-dependent O-glycan elongation. n = 15 in each group. Two-tailed Student’s *t*-test; p = 0.0001 and p = 0.0011. (B) Immunohistochemical staining for α-smooth muscle actin (αSMA), a marker of activated fibroblasts/myofibroblasts, showed markedly increased stromal activation in KCC pancreata at 4 months. At 12 months, αSMA-positive areas were comparable between genotypes, consistent with advanced stromal activation in both tumor-bearing groups at later disease stages. n = 9 KC and KCC at 4 months; n = 5 KC and KCC at 12 months. Two-tailed Student’s *t*-test; p < 0.0001 and p = 0.205. (C) Ki67 staining demonstrated significantly increased proliferative activity in KCC pancreata at 4 months, supporting accelerated expansion of the neoplastic compartment during early tumor progression. n = 10 each. Two-tailed Student’s *t*-test; p < 0.0001. (D) Astra blue staining showed abundant acidic mucin production in KC lesions, whereas KCC tumors displayed reduced Astra blue-reactive acidic mucin-associated glycan structures, consistent with altered mucin maturation and glycosylation upon *Cosmc* deletion (n = 5 each). Two-tailed Student’s *t*-test; p = 0.0002. Representative images are shown for each staining. All images were acquired at 100 × magnification. Scale bar, 200 μm. Data are presented as mean ± SEM.

Desmoplastic stromal remodeling is a defining feature of pancreatic ductal adenocarcinoma (PDAC) and contributes to tumor progression and therapeutic resistance. To determine whether truncated O-glycosylation alters stromal architecture during *Kras*-driven pancreatic tumorigenesis, we analyzed pancreatic sections from KC and KCC mice at 4 and 12 months of age using Masson-Goldner trichrome staining and α-smooth muscle actin (αSMA) immunohistochemistry. At 4 months, KCC pancreata showed pronounced stromal expansion compared with KC controls. Trichrome-positive area was markedly increased in KCC mice relative to KC mice (19.39% vs. 2.37%; p < 0.0001), accompanied by a significant expansion of αSMA-positive regions (13.44% vs. 2.05%; p < 0.0001; Fig. 2A and B). At 12 months, fibrosis remained significantly higher in KCC pancreata (40.65% vs. 28.83%; p = 0.0011), whereas αSMA-positive area no longer differed significantly between genotypes (17.81% vs. 16.67%; p = 0.2048). These findings indicate that *Cosmc* loss is associated with early fibroblast activation and sustained fibrotic remodeling during pancreatic tumor progression.

Consistent with accelerated lesion progression, KCC pancreata also displayed increased proliferative activity at 4 months. Ki67 staining revealed a nearly threefold higher proliferative index in KCC lesions compared with KC controls (74.10% vs. 26.74%; p < 0.0001; Fig. 2C). We next assessed acidic mucin-associated glycan structures using Astra blue staining. KC pancreata contained detectable Astra blue-positive areas, whereas staining was absent in KCC pancreata (5.22% vs. 0%; p = 0.002; Fig. 2D). This difference does not indicate loss of mucin expression but is consistent with altered mucin O-glycosylation and reduced detection of acidic glycan structures following *Cosmc* deletion. Representative immunohistochemical staining for Muc4 and Muc5AC in pancreatic tissues from 12-month-old KC and KCC mice demonstrated positivity in both genotypes (Supplementary Fig. 3 E). Together, these findings show that *Cosmc* loss is associated with increased fibrosis, enhanced proliferation, and remodeling of mucin-associated O-glycans during *Kras*-driven pancreatic neoplastic progression. Pancreatic amylase and pan-cytokeratin staining further demonstrated accelerated acinar loss and acinar-to-ductal metaplasia in KCC compared with KC pancreata at 4 and 12 months (Supplementary Fig. 4A and B). Immune-cell profiling revealed infiltration of CD4⁺ and CD8⁺ T cells and F4/80⁺ macrophages in KC and KCC pancreata, whereas these populations were largely absent from WT and *Cosmc*-KO tissues. CD8⁺ T-cell numbers were highest in KCC pancreata, while macrophage abundance was comparable between KC and KCC mice (Supplementary Fig. 4C).

### Cosmc loss induces robust Tn-like O-glycan expression in pancreatic tumors

**Fig. 3:**
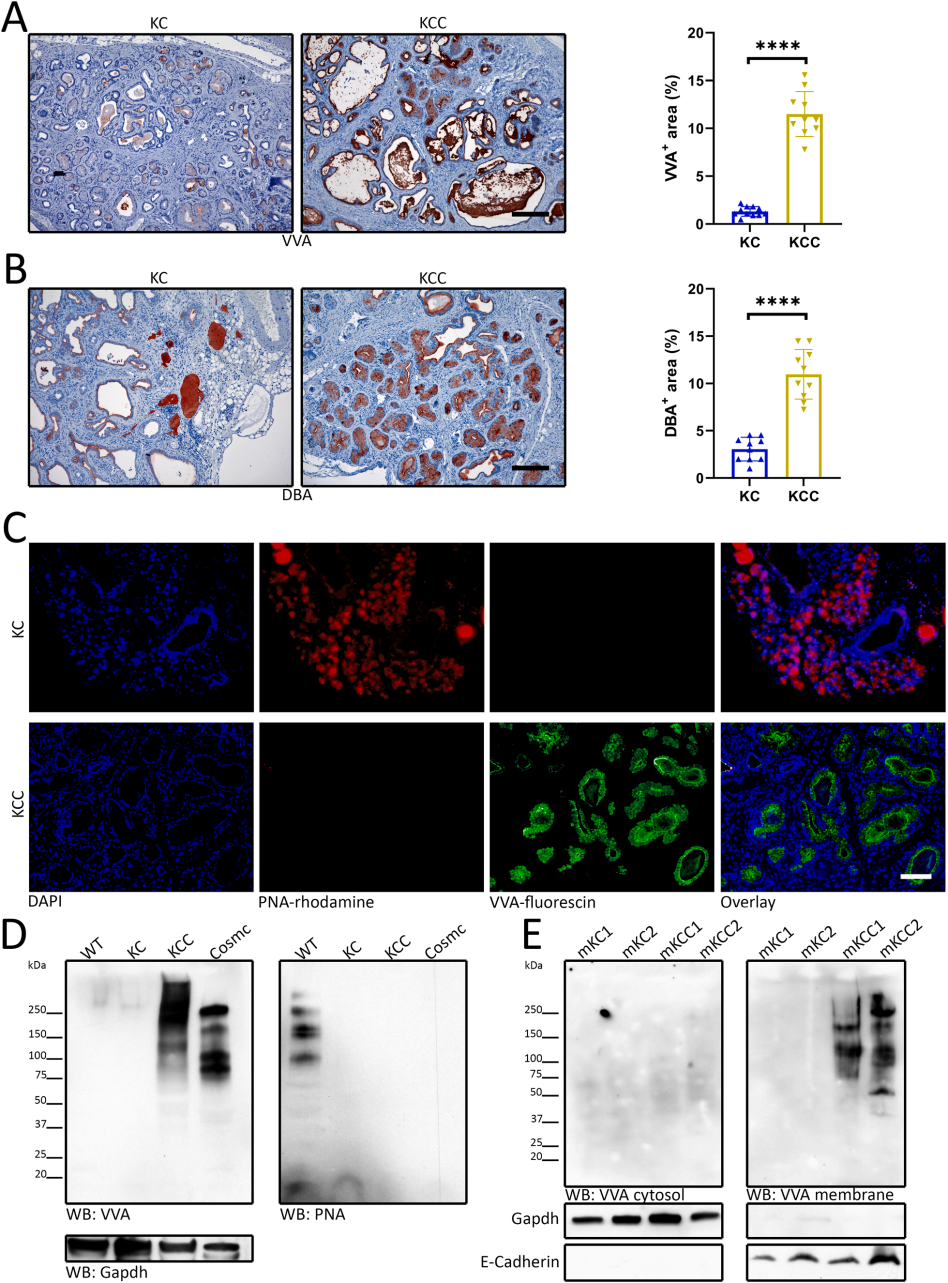
**Lectin-based analyses confirm robust Tn antigen expression after*****Cosmc*****loss in*****Kras*****-driven pancreatic tumors.** To validate the glycosylation phenotype induced by pancreatic epithelial deletion of *Cosmc*, lectin-based histological and biochemical analyses were performed in KC and KCC pancreatic tissues and derived cell lines. (A–B) Lectin histochemistry using *Vicia villosa* agglutinin (VVA), which recognizes terminal α-*N*-acetylgalactosamine residues, and *Dolichos biflorus* agglutinin (DBA), which binds α-GalNAc structures with narrower specificity, revealed markedly increased lectin reactivity in KCC pancreata at 12 months (n = 10 each). Two-tailed Student’s *t*-test; both p < 0.0001. These findings demonstrate accumulation of truncated O-GalNAc glycans/Tn antigen in *Cosmc*-deficient *Kras*-driven pancreatic tumors. (C) Fluorescent lectin staining at 4 months showed reciprocal lectin binding between genotypes: KC pancreata retained peanut agglutinin (PNA) reactivity, consistent with core 1 O-glycan expression, whereas KCC pancreata showed loss of PNA binding and strong VVA positivity, indicating impaired core 1 O-glycan formation and gain of terminal Tn antigen. (D) Lectin blot analysis confirmed genotype-specific glycan remodeling at the protein level, with PNA-reactive glycoproteins detected in WT/KC tissues and VVA-reactive glycoproteins enriched in *Cosmc*-deficient and KCC tissues. (E) Subcellular lectin blotting of KC- and KCC-derived pancreatic cancer cell lines further demonstrated selective enrichment of VVA-reactive glycoproteins in KCC cells, most prominently in membrane-associated fractions, indicating that *Cosmc* loss generates a cell-autonomous, membrane-associated Tn-positive glycoprotein phenotype. Representative images are shown. Images in A–B were acquired at 100 × magnification; scale bar, 200 μm. Images in C were acquired at 200 × magnification; scale bar, 100 μm. Data are presented as mean ± SEM.

To determine whether pancreatic *Cosmc* deletion induces truncated O-glycans *in vivo*, we performed lectin histochemistry on 12-month-old KC and KCC pancreatic tumors using *Vicia villosa* agglutinin (VVA), which recognizes terminal GalNAc residues. VVA staining was minimal in KC tumors but markedly increased in KCC tissues (1.31% versus 10.90% positive area; p < 0.0001; Fig. 3A), consistent with accumulation of Tn-like O-glycan structures following *Cosmc* loss. *Dolichos biflorus* agglutinin (DBA), which displays more restricted α-GalNAc specificity, showed a similar increase in KCC tumors compared with KC controls (3.06% versus 10.97%; p < 0.0001; Fig. 3B). Thus, *Kras*-driven tumors retain low basal levels of terminal GalNAc reactivity, whereas *Cosmc* deletion strongly amplifies this glycophenotype. Dual fluorescent lectin staining of 4-month-old pancreata further confirmed the shift from elongated core 1 O-glycans to truncated GalNAc-terminated structures. Peanut agglutinin (PNA), which recognizes core 1 O-glycans, labeled KC tissue but was largely absent in KCC pancreata, whereas VVA staining was absent in KC and strongly induced in KCC tissue (Fig. 3C). Lectin blotting of pancreatic lysates supported these findings at the protein level: PNA binding was restricted to WT and KC tissues, while VVA selectively detected glycoproteins in KCC and *Cosmc*-KO pancreata (Fig. 3D).

To assess the subcellular distribution of this glycophenotype, we analyzed cytoplasmic and membrane fractions from KC- and KCC-derived pancreatic cancer cell lines. VVA reactivity was restricted to KCC cells and was most prominent in membrane-associated proteins, with weaker cytoplasmic signals and line-specific glycoprotein patterns (Fig. 3E and F). These data establish that *Cosmc* loss induces a robust and heterogeneous Tn-like glycoprotein signature in pancreatic tumors and derived cancer cells, with prominent enrichment at the membrane compartment.

### Cosmc deletion induces cell-cycle-associated transcriptional reprogramming in Kras-driven PDAC cells

**Fig. 4:**
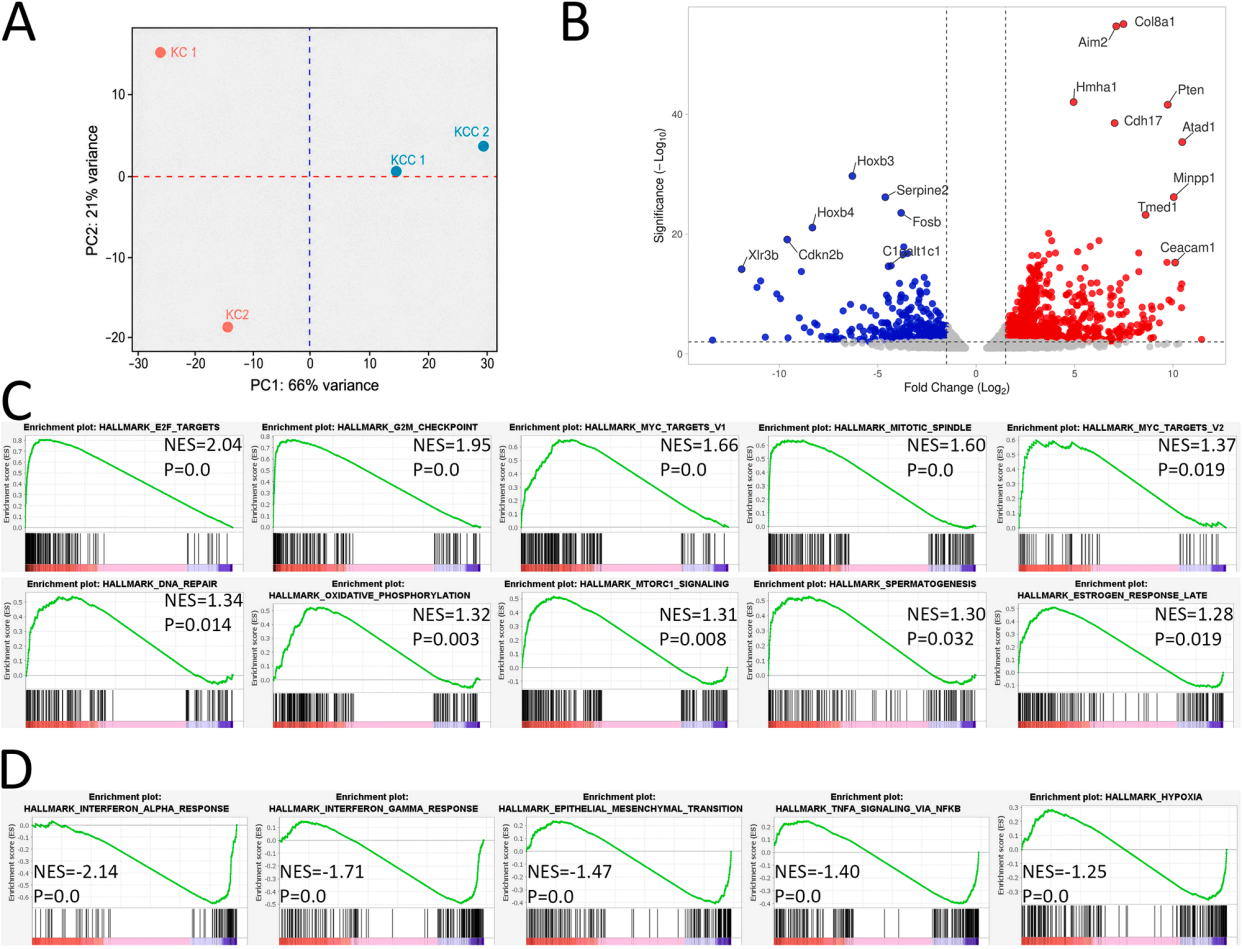
***Cosmc*****loss induces cell-cycle-associated transcriptional remodeling in*****Kras*****-driven pancreatic cancer cells.** RNA sequencing was performed on pancreatic cancer cell lines derived from KC and KCC mice to determine whether loss of *Cosmc* alters tumor cell-intrinsic transcriptional programs. (A) Principal component analysis showed separation of KC and KCC cell lines along PC1, which accounted for 66% of the total variance, indicating genotype-associated transcriptomic divergence despite inter-line variability. (B) Volcano plot of differentially expressed genes in KCC versus KC cells. Overall, 912 genes were significantly altered, with upregulation of genes including *Col8a1, Aim2, Hmha1, Pten, Cdh17, Atad1, Minpp1, Tmed1*, and *Ceacam1*, and downregulation of genes including *Xlr3b, Cdkn2b, Hoxb3, Hoxb4, Serpine2, Fosb*, and *C1galt1c1*. (C) Gene set enrichment analysis revealed positive enrichment of cell-cycle- and proliferation-associated Hallmark programs in KCC cells, including E2F targets, G2/M checkpoint, mitotic spindle, MYC targets, DNA repair, oxidative phosphorylation, and MTORC1 signaling, indicating activation of transcriptional programs linked to cell-cycle progression and metabolic activity. (D) Conversely, several inflammatory, stress-response, and microenvironment-associated Hallmark gene sets were negatively enriched in KCC cells, including interferon-α response, interferon-γ response, epithelial–mesenchymal transition, TNFα signaling via NF-κB, and hypoxia. Together, these data indicate that *Cosmc* loss reshapes *Kras*-driven pancreatic cancer cells toward a cell-cycle-associated transcriptional state while reducing selected immune-, stromal-, and stress-associated programs under standard culture conditions. Differentially expressed genes were defined by adjusted p < 0.05 and log₂ fold change ≥ 1.

To define the tumor cell-intrinsic consequences of *Cosmc* loss, we performed RNA sequencing of pancreatic ductal cell lines derived from KC and KCC mice (Supplementary Fig. 5). Principal component analysis separated KC and KCC cells along the first principal component, which accounted for 66% of the total variance, indicating genotype-associated transcriptional divergence despite inter-line heterogeneity within each group (Fig. 4A). Differential expression analysis identified 912 genes altered in KCC cells, comprising 596 upregulated and 316 downregulated transcripts. Among the most prominently increased genes were *Col8a1, Aim2, Hmha1, Pten, Cdh17, Atad1, Minpp1, Tmed1*, and *Ceacam1*, whereas *Xlr3b, Cdkn2b, Hoxb3, Hoxb4, Serpine2, Fosb*, and *C1galt1c1* were reduced, confirming efficient loss of the *Cosmc*-dependent glycosylation program (Fig. 4B). Gene set enrichment analysis revealed a pronounced shift toward cell-cycle- and growth-associated transcriptional programs in KCC cells. Hallmark pathways enriched in KCC included E2F targets, G2M checkpoint, MYC targets, mitotic spindle, DNA repair, oxidative phosphorylation, and mTORC1 signaling (Fig. 4C). These signatures indicate that *Cosmc* deletion reinforces transcriptional networks linked to cell-cycle entry, biosynthetic metabolism, mitochondrial activity, and genome maintenance in the context of oncogenic *Kras*.

In contrast, inflammatory and microenvironment-associated programs were relatively reduced in KCC cells. Interferon-α response, interferon-γ response, epithelial–mesenchymal transition, TNF-α signaling via NF-κB, and hypoxia were negatively enriched in KCC compared with KC cells (Fig. 4D). This attenuation of immune-inflammatory and stress-associated transcriptional signatures in isolated tumor cells contrasts with the pronounced stromal remodeling observed in KCC tumors *in vivo*, suggesting that *Cosmc* loss may differentially affect tumor cell-autonomous programs and tissue-level tumor-microenvironment interactions.

### Cosmc loss defines an extracellular matrix-associated GalNAc glycoproteome in Kras-driven PDAC

**Fig. 5:**
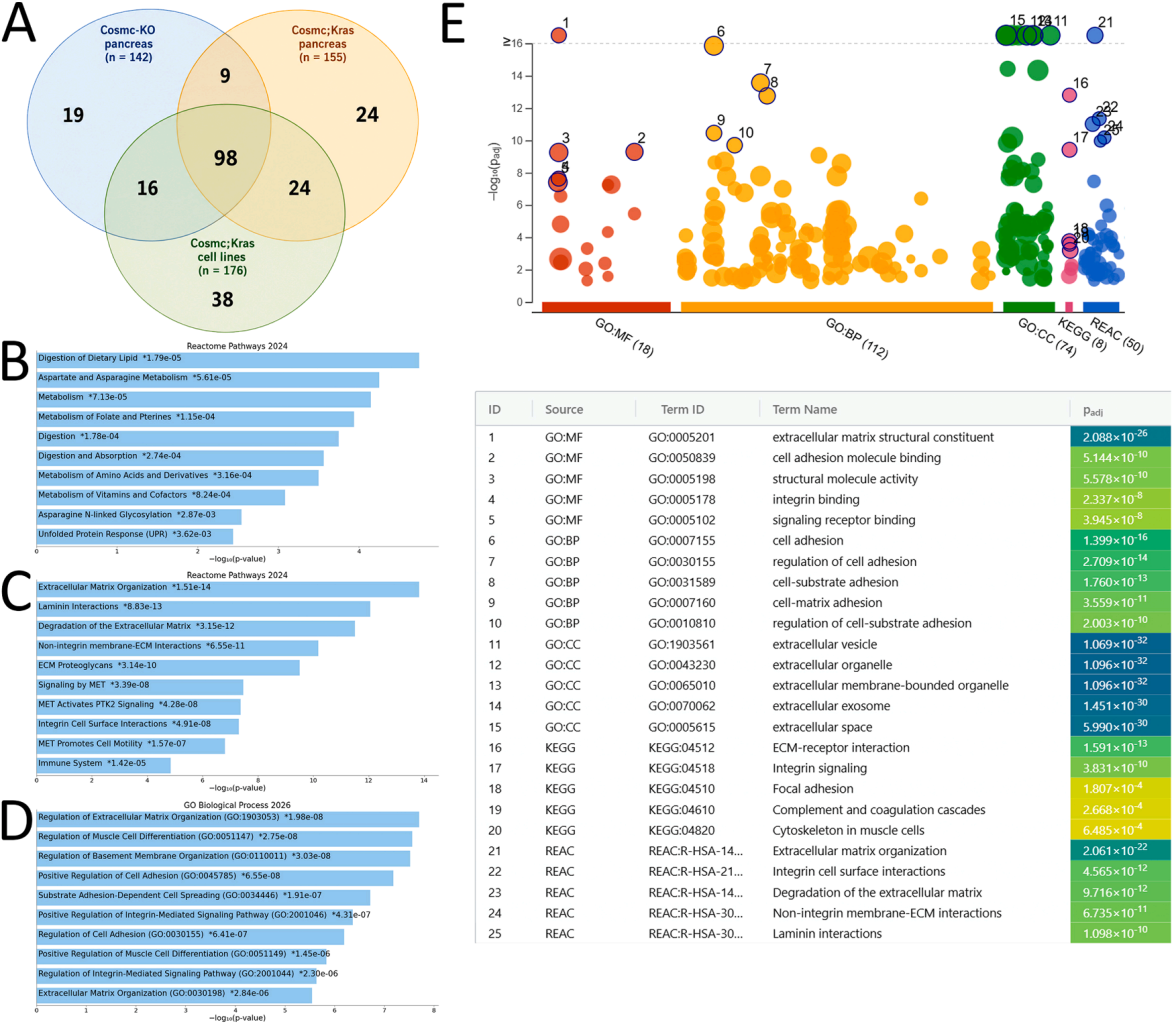
***Cosmc*****loss defines an extracellular matrix-associated GalNAc glycoproteome in*****Kras*****-driven pancreatic cancer.** VVA lectin-enrichment proteomics was used to profile GalNAc-associated glycoproteins in *Cosmc*-KO pancreas, KCC pancreatic tumors, and KCC-derived pancreatic cancer cell lines. (A) Overlap analysis identified 142 VVA-enriched proteins in *Cosmc*-KO pancreas, 155 in KCC pancreas, and 176 in KCC cell lines, including 98 proteins shared across all three datasets. (B) Reactome enrichment of *Cosmc*-KO pancreas proteins highlighted digestive, metabolic, and unfolded protein response-associated pathways, consistent with remodeling of the exocrine pancreatic glycoproteome. (C) In KCC tumors, VVA-enriched proteins were enriched for extracellular matrix organization, laminin interactions, ECM degradation, ECM proteoglycans, MET signaling, and cell migration-related pathways. (D) GO biological process analysis of KCC-derived cell lines similarly highlighted extracellular matrix organization, basement membrane organization, cell adhesion, substrate-dependent cell spreading, and integrin-mediated signaling, indicating a tumor cell-intrinsic adhesion- and matrix-associated glycophenotype. (E) Integrated enrichment of the conserved VVA-enriched protein set confirmed overrepresentation of extracellular matrix structural constituents, cell adhesion molecule binding, integrin binding, extracellular vesicle and extracellular space compartments, ECM–receptor interaction, focal adhesion, integrin signaling, ECM degradation, non-integrin membrane–ECM interactions, and laminin interactions. Together, these data define a conserved extracellular matrix-associated GalNAc glycoproteome linked to *Cosmc* loss in *Kras*-driven pancreatic cancer.

To define the protein landscape associated with truncated O-glycosylation, we performed VVA lectin-enrichment proteomics in three *Cosmc*-deficient settings: *Cosmc*-KO pancreas, KCC pancreatic tumors and KCC-derived PDAC cell lines. This identified 142, 155 and 176 VVA-enriched proteins, respectively, with 98 proteins shared across all three datasets (Fig. 5A). Additional overlap was observed between KCC tumors and KCC cell lines, whereas each dataset also contained context-specific proteins, indicating both conserved and tissue-dependent remodeling of the GalNAc-associated proteome. Pathway analysis revealed distinct but convergent biological programs. *Cosmc*-deficient pancreatic tissue was enriched for digestive and metabolic pathways, including lipid digestion, amino acid metabolism, folate and vitamin/cofactor metabolism, and the unfolded protein response (Fig. 5B). In contrast, the PDAC-associated VVA-enriched proteome was dominated by extracellular matrix and stromal-remodeling pathways, including extracellular matrix organization, laminin interactions, extracellular matrix degradation, non-integrin membrane-ECM interactions, MET and TGFβ-related signaling, MMP activity and immune-system-associated processes (Fig. 5C). These pathways paralleled the histological phenotype of KCC tumors, which showed accelerated fibrosis and stromal expansion. Gene ontology analysis further resolved this signature as an adhesion- and matrix-centered program. Enriched biological processes included regulation of extracellular matrix organization, basement membrane organization, cell adhesion, substrate-dependent cell spreading and integrin-mediated signaling (Fig. 5D). Integrated GO, KEGG and Reactome analysis confirmed extracellular matrix structural constituents, cell adhesion molecule binding, integrin binding, extracellular vesicle/exosome compartments, focal adhesion, ECM-receptor interaction, integrin signaling and laminin interactions among the most significant terms (Fig. 5E).

Thus, *Cosmc* loss establishes a conserved GalNAc-associated protein signature across pancreatic tissue, KCC tumors and KCC-derived cell lines. Rather than affecting isolated glycoproteins, truncated O-glycosylation is associated with coordinated remodeling of extracellular matrix, adhesion, integrin/laminin and vesicle-associated networks, identifying molecular programs associated with stromal remodeling following *Cosmc* loss.

## Discussion

Aberrant glycosylation is increasingly recognized as a functional layer of tumor biology that acts alongside genetic and transcriptional alterations [33,34]. Defective mucin-type O-glycan elongation, marked by accumulation of the truncated Tn (CD175) and sialyl-Tn (sTn, CD175s) antigens, occurs across epithelial malignancies, including pancreatic ductal adenocarcinoma (PDAC) [[35], [36], [37], [38], [39]]. Here, we show that truncated O-glycans are frequent in human PDAC and that genetic disruption of *Cosmc*-dependent core 1 O-glycan maturation accelerates *Kras*-driven pancreatic tumor progression *in vivo*. These findings identify defective O-glycan elongation as an active modifier of pancreatic tumor evolution in the presence of oncogenic *Kras*.

In human PDAC, Tn and sTn were frequently expressed, with Tn associated with tumor stage. Since these correlative data could not establish causality, we generated a pancreas-specific *Cosmc*-deficient *Kras*-driven mouse model. *Kras*-driven genetically engineered models remain central to studying PDAC initiation and progression, as oncogenic *KRAS* mutations occur in >90% of human PDAC and represent early initiating events [[19], [20], [21], [22], [23], [24]]. In this context, our KCC model demonstrates that *Cosmc* loss accelerates *Kras*-driven pancreatic lesion progression without requiring additional *Trp53* mutation. Targeted disruption of *Cosmc* induced robust endogenous Tn antigen expression and shifted *Kras*-driven pancreatic disease toward earlier and more extensive local progression. KCC pancreata showed accelerated PanIN development, increased tissue proliferation, depletion of acidic mucins and pronounced stromal remodeling. However, neither KC nor KCC mice developed widespread metastatic disease or a uniformly lethal phenotype within the analyzed time frame. Consistent with this relatively indolent disease course, no spontaneous or tumor-associated mortality and no distant metastatic dissemination were observed during follow-up of up to 24 months. Thus, *Cosmc* loss does not convert the KC model into an aggressive metastatic PDAC model but acts as a cooperating modifier that enhances local tumor progression and tissue remodeling in a *Kras*-mutant setting. This phenotype is distinct from that of pancreas-specific *Cosmc* deletion alone. In the absence of mutant *Kras, Cosmc* loss primarily produces an acinar secretory phenotype characterized by impaired digestive-enzyme function and secretion, zymogen-granule accumulation, exocrine insufficiency, age-dependent pancreatic atrophy, and secondary disturbances in glucose homeostasis. No spontaneous pancreatic neoplastic phenotype was observed in the previously characterized model [18]. By contrast, in the presence of oncogenic *Kras, Cosmc* loss accelerates PanIN progression, proliferation, fibrosis, and pancreatic tissue remodeling. Together, these observations indicate that truncated O-glycosylation is not independently tumor-initiating in this model but acts as a cooperating modifier of *Kras*-driven neoplastic progression.

This interpretation extends previous work showing that disruption of core 1 O-glycosylation promotes pancreatic cancer progression in more advanced genetic contexts. Deletion of *C1galt1*/T-synthase in KPC mice induces truncated O-glycan expression, accelerates PanIN development and promotes early metastasis [26]. By contrast, our study examines *Cosmc* loss in a *Kras*-only background, showing that defective core 1 O-glycan maturation can cooperate with oncogenic *Kras* before acquisition of additional strong tumor suppressor alterations. Reports of frequent *C1GALT1* overexpression in gastrointestinal cancers [40,41] further emphasize that regulation of this pathway is complex and may involve both loss-of-function events and compensatory changes in glycan biosynthesis.

A central implication of our study is that truncated O-glycosylation reshapes the molecular state of *Kras*-driven pancreatic tumors. Transcriptomic and proteomic profiling revealed genotype-associated reprogramming, while VVA-enriched glycoproteomics identified changes linked to extracellular matrix organization, focal adhesion, cytoskeletal regulation, stress adaptation and metabolic remodeling [[42], [43], [44]]. These molecular changes align with the stromal phenotype observed *in vivo* and suggest that *Cosmc* loss affects not only individual glycan epitopes, but the broader glycoprotein landscape through which tumor cells interact with their microenvironment. In this model, truncated O-glycans may promote PDAC progression by altering epithelial glycoprotein states, matrix-associated proteins and adhesion-related programs that shape stromal organization and tissue architecture.

Several studies support a broader role for *COSMC* or *C1GALT1* disruption in pancreatic and gastrointestinal cancer. *COSMC* promoter hypermethylation contributes to truncated O-glycan expression in PDAC [11], while altered COSMC/C1GALT1 function has been linked to proliferation, adhesion, invasiveness, epithelial–mesenchymal transition, plasticity and stem-like traits [[45], [46], [47]]. Our previous work further showed that *COSMC* knockdown affects AKT/mTOR and RAS/MAPK signaling, with consequences for motility and apoptosis resistance [10,48]. Together with the present *in vivo* data, these studies support defective O-glycan maturation as a modifier of tumor cell behavior and tumor–microenvironment interactions.

Nevertheless, aberrant O-glycosylation alone appears insufficient to initiate pancreatic neoplasia. Exocrine pancreas-specific *Cosmc* deletion induces Tn antigen expression and exocrine dysfunction without spontaneous tumor formation [18]. Similarly, pancreas-specific GalNT2 overexpression causes marked structural and functional abnormalities without neoplastic progression [46], and forced Tn antigen expression has been associated with chronic inflammation and fibrosis in the absence of malignancy [49]. Thus, glycan truncation disrupts pancreatic homeostasis but requires an oncogenic context, such as mutant *Kras*, to promote neoplastic progression.

Several limitations should be acknowledged. Although transcriptomic and VVA-enriched proteomic analyses identified molecular programs associated with *Cosmc* loss, they do not define which individual glycoproteins causally drive tumor acceleration. Moreover, VVA enrichment detects terminal GalNAc-associated proteins but does not resolve site-specific O-glycosylation or distinguish direct glycoprotein targets from associated complexes. Because transcriptomic profiling was performed in tumor-derived cell lines, stromal and immune interactions *in vivo* are only partially captured. Future site-resolved O-glycoproteomics, spatial analyses, and functional perturbation of candidate ECM or adhesion-associated targets will be required to define how truncated O-glycosylation promotes pancreatic tumor-stroma remodeling.

In summary, we establish a pancreas-specific *Cosmc*-deficient *Kras*-driven mouse model that develops endogenous Tn antigen O-glycan expression and accelerated pancreatic neoplastic progression. Together with human PDAC tissue analysis, transcriptomic profiling, and VVA-enriched glycoproteomics, our findings identify *Cosmc*-dependent O-glycan maturation as a modifier of *Kras*-driven pancreatic carcinogenesis. Our findings suggest that defective core 1 O-glycan maturation is associated with altered epithelial glycoprotein states and stromal architecture during *Kras*-driven pancreatic tumor evolution.

## Funding

This research was funded by the German Research Foundation (DFG) grant number 275533756 to GW-E.

## Ethics approval and consent to participate

The use of archived diagnostic leftover human tissue samples for the construction of tissue microarrays and subsequent research analysis, as well as the analysis of associated patient data, was approved by the Ethics Committee of Hamburg (approval numbers WF-049/09 and PV3652). The breeding and euthanasia of mice for scientific research were approved by the local regulatory authority, *Behörde für Justiz und Verbraucherschutz* (ORG_616, ORG_753, and ORG_754). All procedures involving human and animal materials were conducted in accordance with the relevant guidelines and regulations. The study complies with the ethical standards set forth in the Declaration of Helsinki.

## Consent for publication

All authors have reviewed and approved the final version of the manuscript and consent to its publication.

## Declaration of generative AI and AI-assisted technologies in the manuscript preparation process

During the preparation of this work, the authors used ChatGPT for language editing. The authors reviewed and edited the output as needed and take full responsibility for the content of the published article.

## CRediT authorship contribution statement

**Baris Mercanoglu:** Writing – original draft, Validation, Methodology, Investigation, Formal analysis. **Nina Schraps:** Writing – original draft, Validation, Investigation. **Anastasios D. Giannou:** Validation, Investigation, Formal analysis. **Jingxuan Zhou:** Validation, Investigation, Formal analysis. **Simon Kind:** Validation, Investigation, Formal analysis. **Maya Reinecke:** Validation, Methodology, Investigation, Formal analysis. **Karl-Frederick Karstens:** Validation, Investigation, Formal analysis. **Benjamin Dreyer:** Validation, Methodology, Investigation, Formal analysis, Data curation. **Sönke Harder:** Validation, Methodology, Investigation, Formal analysis, Data curation. **Siwen Zhang:** Validation, Investigation, Formal analysis. **Eleftherios D. Papazoglou:** Validation, Investigation, Formal analysis, Data curation. **Cenap Güngör:** Validation, Investigation, Formal analysis. **Nathaniel Melling:** Validation, Supervision, Formal analysis. **Hartmut Schlüter:** Validation, Supervision, Investigation. **Christoph Wagener:** Validation, Supervision, Resources, Project administration, Conceptualization. **Maximilian Bockhorn:** Validation, Supervision, Project administration, Investigation. **Thilo Hackert:** Supervision, Resources. **Gerrit Wolters-Eisfeld:** Writing – review & editing, Writing – original draft, Visualization, Validation, Supervision, Investigation, Funding acquisition, Formal analysis, Conceptualization.

## Declaration of competing interest

The authors declare no competing interests.

## Appendix Supplementary materials

Image, application 1

Image, application 2

Image, application 3
