# Supplementary material for: Cosmc loss induces truncated O-glycosylation and accelerates Kras-driven pancreatic carcinogenesis
Source: Neoplasia. 2026 Jul 31;80:101347. doi: 10.1016/j.neo.2026.101347 (PMC13453609; doi:10.1016/j.neo.2026.101347)
Supplement: Supplementary file 1 [file mmc1.docx]

**Supplementary Figure 1. Schematic overview of the breeding strategy for generating KC and KCC mouse models.** (A) To generate mice with endogenous pancreatic carcinoma, LSL-Kras^G12D/+^ (B6.129-Krastm4Tyj) mice were crossed with p48-Cre (Ptf1a^tm1(cre)Hnak^) mice to obtain the KC model (LSL-Kras^G12D/+^; Ptf1a^Cre/+^). (B) The KCC model (LSL-Kras^G12D/+^; Cosmc^fl/fl^; Ptf1a^Cre/+^) was established by additional breeding with mice carrying a conditional knockout allele of C1galt1c1 (Cosmc), in which exon 2 is flanked by loxP sites. Cre-mediated recombination under the control of the pancreas-specific Ptf1a promoter induces expression of oncogenic Kras^G12D^ and deletion of Cosmc specifically in pancreatic tissues. All mouse lines were maintained on a C57BL/6J background and bred at the animal facility of the University Medical Center Hamburg-Eppendorf

**B**

**A**

**D**

**C**

**Supplementary Figure 2. *Immunohistochemical analysis of Tn and STn antigen expression in human PDAC.*** (A) Representative TMA images showing negative, weak, and strong staining for Tn (CD175) and STn (CD175s) antigens in FFPE PDAC tissues (100× magnification). (B) Distribution of Tn (n = 163) and STn (n = 186) expression across PDAC cases. Tn was detected in 67% of tumors and correlated with tumor stage (p = 0.0055), particularly in pT2 and pT4 lesions. STn was present in 66% of tumors without stage association. (C–D) Kaplan–Meier analysis revealed no significant correlation between Tn or STn expression and overall survival (p = 0.5583 and p = 0.5572, respectively).

**Supplementary Tab. 1.	Clinicopathological associations of Tn and STn antigen expression in PDAC.** Tn (CD175) and STn (CD175s) expression was assessed by immunohistochemistry in 163 and 186 PDAC samples, respectively, and categorized as negative, weak, or strong. Tn expression was significantly associated with tumor stage (p = 0.0055), indicating stage-dependent regulation. No significant correlations were observed for lymph node status, margin status, relapse, or grade. STn expression showed no significant association with any clinicopathological parameter.

**Supplementary Figure 3. Validation of Cosmc loss and assessment of MAPK signaling in pancreatic tissues and tumor-derived cell lines.** (A) Relative Cosmc mRNA expression in pancreatic tissues from WT, Cosmc-KO, KC, and KCC mice at 4 and 12 months. Data are shown as mean ± SD. **P < 0.01, ****P < 0.0001. (B) Representative Western blot analysis of Cosmc protein expression in the corresponding pancreatic tissues; β-actin served as the loading control. (C) Representative Western blot analysis of phospho-p44/42 MAPK, corresponding to phospho-ERK1/2, and total p38 MAPK in pancreatic tissues from WT, KC, KCC, and Cosmc-KO mice. (D) Representative Western blot analysis of phospho-p44/42 MAPK and total p38 MAPK in independently derived KC and KCC tumor cell lines. Hsp70 served as the loading control in panels C and D. Total p38 MAPK was examined as an additional MAPK-family pathway reference and was not used as the corresponding total-protein normalization control for phospho-p44/42 MAPK. (E) Representative immunohistochemical staining for Muc4 and Muc5AC in pancreatic tissues from 12-month-old KC and KCC mice.

**C**

**Supplementary Fig. 4. Pancreatic amylase expression, acinar-to-ductal metaplasia, and immune-cell characterization.** (A) Representative pancreatic amylase immunohistochemistry using anti-amylase antibody (Abcam, ab199132) in 4-month-old KC and KCC mice. (B) Immunofluorescence staining for pancreatic amylase (red; Abcam, ab199132) and pan-cytokeratin (green; Millipore-Chemicon, AE1/AE3, MAB1612) in KC and KCC pancreata at 4 and 12 months. (C) Quantification of CD4⁺ T cells, CD8⁺ T cells, and F4/80⁺ macrophages in pancreatic sections from WT, KC, KCC, and Cosmc-KO mice. Bars show mean ± SEM; symbols indicate individual animals. One-way ANOVA with multiple-comparison correction; *P < 0.05, **P < 0.01. Scale bars, 100 µm.

**Supplementary Figure 5.** *Molecular and phenotypic characterization of murine PDAC cell lines.* (A) PCR genotyping confirms *Kras^G12D^* activation in both KC and KCC cell lines and *C1galt1c1* (Cosmc) deletion exclusively in KCC, indicating successful Cre-mediated recombination. (B–C) Western blot analysis shows Tn and STn antigen expression in KCC but not KC cells, consistent with loss of core 1 O-glycosylation. Lysates were neuraminidase-treated to unmask Tn epitopes. (D) Brightfield images reveal epithelial morphology in KC and KCC lines, with Panc02 included for comparison (400× magnification).

**Supplementary Fig. 6. Protein-level validation of RNA-seq findings.** Western blot analysis of lysates from KC- and KCC-derived cell lines using antibodies against FN1 (CST, #62601), SERPINH1 (CST, #60448), and COSMC (Thermo Fisher Scientific, #19254-1-AP). β-Actin (Abcam, #ab8227) served as the loading control.

**Supplementary Materials and Methods:**

**Quantitative real-time PCR**

Total RNA was isolated from mouse pancreatic tissue or cultured cells using the RNeasy Mini Kit (Qiagen) according to the manufacturer’s instructions. RNasin Ribonuclease Inhibitor (Promega) was included during RNA preparation to minimize RNase-mediated degradation. RNA was reverse-transcribed using Transcriptor Reverse Transcriptase (Roche) and oligo(dT) primers in accordance with the manufacturer’s protocol.

Quantitative real-time PCR (qRT-PCR) was performed using Maxima SYBR Green/ROX qPCR Master Mix and a LightCycler 480 Real-Time PCR System (Roche). Each reaction contained 0.3 µM forward primer, 0.3 µM reverse primer, and cDNA corresponding to the indicated RNA input. The amplification program consisted of an initial denaturation at 95 °C for 10 min, followed by 45 cycles of denaturation at 95 °C for 15 s and combined annealing and extension at 60 °C for 60 s.

Expression of C1galt1c1, encoding the molecular chaperone Cosmc, was quantified using a commercially validated SYBR Green-compatible primer pair targeting the mouse reference transcript NM_021550. Hypoxanthine phosphoribosyltransferase 1 (Hprt1) was used as the reference gene. The following primer sequences were used:

C1galt1c1 (Cosmc) F: GGACCTCTCACGTCCAAGCCT and R: TGCTTCCAAGCATCACACCCTTC

Hprt1	F: CTGGTTAAGCAGTACAGCCCCAA and R: CGAGAGGTCCTTTTCACCAGC

Quantification cycle values were determined using LightCycler 480 Software, version 1.5 (Roche). All samples were analyzed in technical duplicate. Relative C1galt1c1 expression was normalized to Hprt1 and calculated using the comparative quantification-cycle method (2^−ΔΔCq).

**Immunofluorescence on FFPE tissues**

FFPE tissue sections were deparaffinized in xylene and rehydrated through a graded ethanol series. Antigen retrieval was carried out in retrieval buffer pH 9 by microwave heating (360 W for 2 min, followed by 900 W for 15 min). Sections were allowed to cool naturally at room temperature for 30 min, and regions of interest were marked using an immunohistochemical pen. After PBS washing, sections were permeabilized with 0.3% Triton X-100 in 5% BSA for 30 min and blocked in 5% BSA for 1 h at room temperature. Slides were incubated overnight at 4°C with primary antibodies against CD4, F4/80, and CD8a. Antibodies used were BD Pharmingen Alexa Fluor 488 Rat Anti-Mouse CD4, BD Horizon BV480 Rat Anti-Mouse F4/80, and BD Pharmingen Alexa Fluor 488 Rat Anti-Mouse CD8a. Nuclei were visualized with DAPI and sections were mounted with ProLong Gold Antifade Mountant containing DAPI (Thermo Fisher Scientific). Images were acquired using a Keyence BZ-X1000 microscope under identical settings for all samples.

**Immunofluorescence quantification and statistical testing**

For each marker, immunofluorescence-positive cells were quantified in four representative areas per FFPE tissue section. The mean of these four measurements was calculated for each mouse and used as a single biological replicate for statistical analysis. Group comparisons were performed across WT, KC, KCC, and Cosmc-KO mice using the Kruskal–Wallis test with Dunn’s post hoc multiple-comparisons test. Data are shown as individual biological replicates with mean ± SD, and differences were considered significant at p < 0.05.
